# AMPK Activation Promotes Tight Junction Assembly in Intestinal Epithelial Caco-2 Cells

**DOI:** 10.3390/ijms20205171

**Published:** 2019-10-18

**Authors:** Séverine Olivier, Jocelyne Leclerc, Adrien Grenier, Marc Foretz, Jérôme Tamburini, Benoit Viollet

**Affiliations:** Université de Paris, Institut Cochin, CNRS UMR8104, INSERM U1016, F-75014 Paris, France; severine.olivier@inserm.fr (S.O.); jocelyne.leclerc@inserm.fr (J.L.); adrien.grenier@aphp.fr (A.G.); marc.foretz@inserm.fr (M.F.); jerome.tamburini@inserm.fr (J.T.)

**Keywords:** AMPK, direct AMPK activator, calcium switch, Caco-2 cells, intestinal barrier function, tight junction, trans-epithelial electrical resistance, paracellular permeability

## Abstract

The AMP-activated protein kinase (AMPK) is principally known as a major regulator of cellular energy status, but it has been recently shown to play a key structural role in cell-cell junctions. The aim of this study was to evaluate the impact of AMPK activation on the reassembly of tight junctions in intestinal epithelial Caco-2 cells. We generated Caco-2 cells invalidated for AMPK α1/α2 (AMPK dKO) by CRISPR/Cas9 technology and evaluated the effect of the direct AMPK activator 991 on the reassembly of tight junctions following a calcium switch assay. We analyzed the integrity of the epithelial barrier by measuring the trans-epithelial electrical resistance (TEER), the paracellular permeability, and quantification of zonula occludens 1 (ZO-1) deposit at plasma membrane by immunofluorescence. Here, we demonstrated that AMPK deletion induced a delay in tight junction reassembly and relocalization at the plasma membrane during calcium switch, leading to impairments in the establishment of TEER and paracellular permeability. We also showed that 991-induced AMPK activation accelerated the reassembly and reorganization of tight junctions, improved the development of TEER and paracellular permeability after calcium switch. Thus, our results show that AMPK activation ensures a better recovery of epithelial barrier function following injury.

## 1. Introduction

Epithelial cells are essential to protect organisms from the external environment. One of their main characteristics is to form a physical barrier thanks to a robust network of junctional proteins that closely link the cells between them and maintain their polarity. Cell-cell contacts involve tight junctions (zonula occludens proteins, occludins, claudins) and adherens junctions (catenins, cadherins). These proteins located at the apical part of epithelial cells regulate the paracellular permeability [1] and form multimolecular complexes where actin microfilaments can attach [2]. Alterations of epithelial barriers and loss of cell polarity are associated with various disorders like cancer development [3], inflammatory bowel diseases [4], or renal injury [5].

The AMP-activated protein kinase (AMPK) is an ubiquitous serine/threonine kinase principally known as an energy sensor at cellular level. It is a heterotrimeric protein composed of a α catalytic subunit and β and γ regulatory subunits existing as multiple isoforms in vertebrates [6]. In response to metabolic stresses where cellular energy balance is compromised, such as during ischaemia, hypoxia, or muscle contraction, AMP/ATP and ADP/ATP ratios are increased which leads to AMPK activation [7]. Binding of AMP on γ subunit enables phosphorylation of AMPK on Thr-172 by upstream kinase Liver Kinase B1 (LKB1) which in turn activates AMPK that phosphorylates downstream targets involved in various metabolic processes to restore cellular energy balance [8]. Ca^2+^/calmodulin-activated protein kinase kinase β (CaMKKβ) is also able to phosphorylate AMPK in response to an increase in intracellular calcium, thus independently of the variation of AMP and ADP concentrations [9]. Furthermore, there exist various natural and pharmacological activators that act via different mechanisms: allosteric activation, phosphorylation of Thr-172, or protection against dephosphorylation of Thr-172 [10].

In addition to its best known metabolism functions, AMPK has been involved in the process of tight junctions’ assembly in epithelial and endothelial cells under stress conditions, highlighting a novel structural function. This role has been first demonstrated in renal Madin-Darby Canine Kidney (MDCK) cells where AMPK inhibition impaired tight junctions reassembly following a calcium switch challenge, whereas AMPK activation facilitated tight junctions assembly [11,12]. Following this breakthrough, other studies have shown the importance of AMPK in the regulation of epithelial and endothelial barriers in lung [13], heart [14] and brain [15].

The beneficial role of AMPK in intestinal health has received much attention in the recent years. Some studies using mouse models that mimic inflammatory bowel diseases showed a correlation between the decrease in intestinal AMPK activity and colitis development [16], and that metformin, an indirect AMPK activator, can protect against epithelial injuries [17,18]. Furthermore, the study of the effects of prebiotics and short chain fatty acids (SCFAs) produced by commensal bacteria on intestinal barrier has become a focus of interest in the last few years. Notably, it has been reported in human colon carcinoma Caco-2 cells that butyrate accelerated the assembly of tight junctions via the activation of AMPK [19,20], suggesting some interactions between AMPK and intestinal microbiota for the regulation of intestinal barrier function. All these previously reported studies strongly support a mechanism involving AMPK-dependent regulation of tight junction assembly, albeit the use of the AMPK inhibitor compound C, which has questionable selectivity [21], and widely-used indirect pharmacological AMPK activators, which have known off-target effects [22]. Thus, the significance of AMPK in the maintenance of intestinal barrier remains unclear.

Here, the aim of the present study was to evaluate the role of AMPK in the reassembly of tight junctions during a calcium switch assay in monolayer of Caco-2 cells by using novel genetic and pharmacological approaches. Notably we examined the effect of a specific and direct AMPK activator 991 on the assembly of tight junction proteins in AMPK-deficient Caco-2 cells generated by CRISPR/Cas9 system. Our study suggests that AMPK represents a druggable target to restore intestinal barrier integrity.

## 2. Results

### 2.1. Deletion of both AMPKα1 and AMPKα2 is Necessary to Abrogate AMPK Signaling in Caco-2 Cells

**Figure 1:**
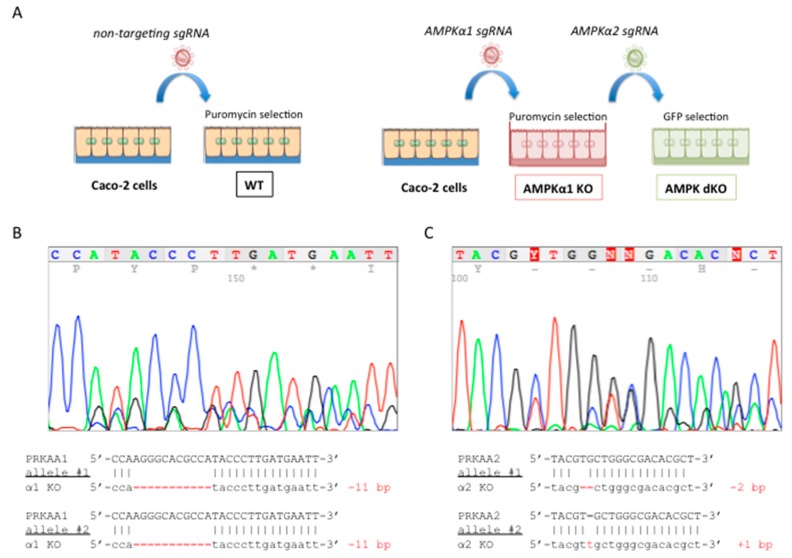
Generation and characterization of AMPKα1/α2-deficient Caco-2 cells. (**A**) Experimental workflow for genome engineering of colon carcinoma Caco-2 cells. A sequential procedure was used to target first *PRKAA1* gene encoding AMPKα1 and then *PRKAA2* gene encoding AMPKα2. Cells expressing *PRKAA1*-targeting sgRNAs were first selected on puromycin resistance and then cells on an AMPKα1 knockout background expressing *PRKAA2*-targeting sgRNAs were selected on green fluorescent protein (GFP) expression. Control cells were generated by using a non-targeting sgRNA and were selected on puromycin resistance. (**B**) Sequencing analysis of *PRKAA1* CRISPR alleles shows that both alleles were modified by deletion of 11 bp, resulting in premature stop codons. (**C**) Sequencing analysis of *PRKAA2* CRISPR alleles shows that one allele displayed a deletion of 2 bp and the second allele an insertion of 1 bp. All these alleles result in premature stop codons.

To examine the role of AMPK in tight junction assembly in intestinal epithelial cells, we invalidated the two catalytic AMPKα1 and AMPKα2 subunits in human colon carcinoma Caco-2 cells by genome editing with the CRISPR/Cas9 system. We used a sequential approach targeting first the *PRKAA1* gene encoding the catalytic AMPKα1 subunit and then the *PRKAA2* gene encoding the catalytic AMPKα2 subunit in single-cell clones, as previously described [23] (Figure 1A). The CRISPR-Cas9 system introduced insertion deletion (indel) mutations in the target sites of *PRKAA1* and *PRKAA2* genes, resulting in premature stop codons (Figure 1B,C).

**Figure 2:**
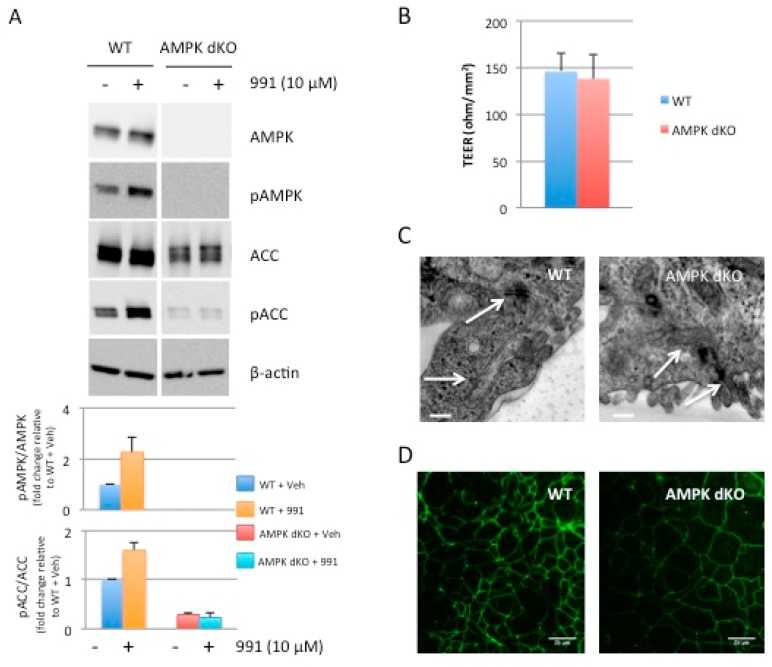
Effect of AMPK deletion on tight junction integrity at steady-state. (**A**) Whole cell lysates of WT and AMPK dKO Caco-2 cells treated with 10 µM 991 for 10 min were analyzed for total and phospho(p)-AMPK and -ACC at Thr-172 and Ser-79, respectively. Expression of β-actin served as loading control. Lower panels represent ratios of pAMPK:AMPK and pACC:ACC from quantification of immunoblot images. (**B**) Variation of trans-epithelial electrical resistance (TEER) in polarized confluent WT and AMPK dKO Caco-2 cells. Cells were grown on Transwell filters for 3 weeks and TEER was measured in WT and AMPK dKO Caco-2 cells. Data represent means ± SD (*n* = 3). (**C**) Transmission electron micrograph of WT and AMPK dKO Caco-2 cells at steady-state. Sections of monolayers of postconfluent stationary cells grown on Transwell filters. Arrows indicate cell-cell junctions. High magnification of intercellular spaces with distinguishable tight junctions are shown. Scale Bar: 200 nm. (**D**) Representative immunostaining of ZO-1 in WT and AMPK dKO Caco-2 cells at steady-state. Scale bar: 25 µm.

Although the catalytic subunit AMPKα1 is predominantly expressed in Caco-2 cells [24], deletion of both AMPK catalytic subunits (AMPKα1 and AMPKα2) was necessary to fully abolish AMPK signaling [23]. Notably, in AMPKα1-deficient (AMPKα1 KO) Caco-2 cells, expression of the non-deleted AMPKα2-isoform was markedly increased when compared to control (WT) cells treated with a non-targeting small guide RNA (sgRNA) (Supplementary Figure S1A). As a result, while activation of AMPK with the direct pan-AMPK pharmacological activator 991 [25] in AMPKα1 KO cells triggered phosphorylation of acetyl CoA carboxylase (ACC) at Ser-79, a well-established target of AMPK (Supplementary Figure S1B), this was completely abolished in double AMPKα1/AMPKα2-deficient (AMPK dKO) Caco-2 cells compared to WT cells (Figure 2A). Taken together, these findings demonstrate that we generated a Caco-2 cell line completely devoid of AMPK activity.

### 2.2. Disruption of AMPK in Caco-2 cells does not Alter the Integrity of Tight Junction at Steady-State

To investigate the effect of AMPK deletion on tight junction assembly, we first measured trans-epithelial electrical resistance (TEER) in monolayers of WT and AMPK dKO Caco-2 cells grown on Transwell filters in normal culture medium for 3 weeks. We found that TEER was similar in polarized confluent WT and AMPK dKO cells (Figure 2B). These findings provide evidence that AMPK is not required for the long-term maintenance of functional tight junction. Consistently, no obvious difference in tight junction morphology could be observed by transmission electron microscopy analysis of WT and AMPK dKO cells at steady-state (Figure 2C), nor in ZO-1 location at plasma membrane analyzed by immunofluorescence (Figure 2D).

### 2.3. Deletion of AMPK Prevents Calcium-Induced Reassembly of Tight Junctions in Caco-2 Cells

**Figure 3:**
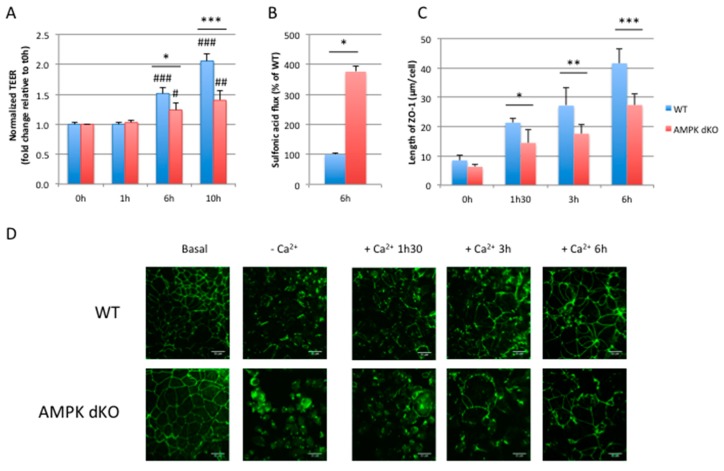
AMPK disruption delays barrier integrity recovery after calcium switch. (**A**) Time course of TEER development in WT and AMPK dKO Caco-2 cells subjected to a calcium switch. Cells grown on Transwell filters were incubated in calcium-free medium for 16 hours and switched to normal calcium medium. TEER was measured at the indicated time points after calcium switch and is given as fold change relative to the value in calcium-free medium at 0 h time point (0 h) (**B**) Paracellular permeability of 0.4 kDa FITC-sulfonic acid in WT and AMPK dKO Caco-2 monolayers subjected to calcium switch. Flux of FITC-sulfonic acid was measured at 6 h time point (6 h) and is given as the percentage of WT value. (**C**) Quantification of ZO-1 deposition at cell-cell junction after calcium switch at the indicated time points in WT and AMPK dKO Caco-2 cells. (**D**) Representative immunostaining of ZO-1 in WT and AMPK dKO Caco-2 cells at steady-state (basal), incubated in calcium-free medium for 16 hours (-Ca^2+^) or subjected to calcium switch (+Ca^2+^) for indicated time. Scale bar: 25 µm. Data represent means ± SD for three independent experiments (*n* = 3). * *p* < 0.05; ** *p* < 0.01; *** *p* < 0.005 versus AMPK dKO cells at the same time point. # *p* <0.05; ## *p* < 0.01; ### *p* < 0.005 versus 0 hour time point (0 h) for the same genotype.

Intercellular junctions between epithelial cells are dependent on extracellular calcium concentrations [26]. Low concentrations of extracellular Ca^2+^ disrupt intercellular junctions, and the restoration of Ca^2+^ concentrations induces the deposition of junction proteins to the plasma membrane and triggers junction assembly. When WT Caco-2 cells are switched from calcium-free to calcium-containing culture medium (calcium switch experiment), TEER is increased over time reflecting the reassembly of tight junctions and restoration of the paracellular barrier function (Figure 3A). However, in AMPK dKO cells, the development of TEER was delayed upon readdition of calcium (Figure 3A), suggesting a role of AMPK in the process of tight junctions reassembly. To further investigate the role of AMPK in the establishment of intestinal barrier integrity, we measured paracellular permeability in WT and AMPK dKO Caco-2 cell monolayers after the calcium switch assay. We observed that AMPK dKO cells displayed a 4-fold increased paracellular flux to 0.4 kDa FITC-conjugated sulfonic acid compared to WT cells, suggesting major defaults in intestinal barrier integrity associated with the delay in TEER development (Figure 3B). Next, we monitored the location of ZO-1 by immunostaining after the calcium switch challenge, and we quantified ramifications of ZO-1 deposit at plasma membrane (Figure 3C). Calcium withdrawal induced the relocation of ZO-1 in the cytoplasm in both WT and AMPK dKO cells (Figure 3D). However, upon readdition of calcium, ZO-1 deposit at plasma membrane was less important in AMPK dKO cells compared to WT cells at 1 h 30 min, 3 h and 6 h, indicating a delay in the reorganization of tight junction proteins in the absence of AMPK (Figure 3C,D). Altogether, our data show that the absence of AMPK in Caco-2 cells induces a delay in the reassembly of tight junctions during the calcium switch assay.

### 2.4. Acute Pharmacological Inhibition of AMPK Prevents Calcium-Induced Reassembly of Tight Junctions in Caco-2 Cells

**Figure 4:**
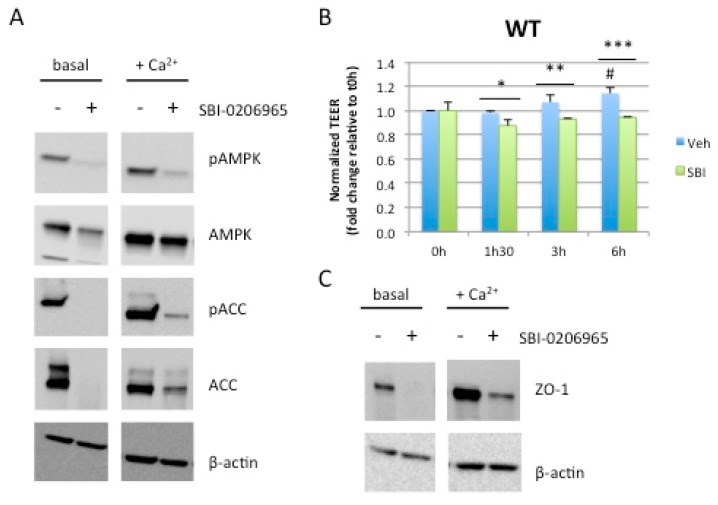
Acute pharmacological inhibition of AMPK prevents establishment of TEER after calcium switch. (**A**) Effect of SBI-0206965 on AMPK signaling. WT Caco-2 cells at steady-state (basal) or subjected to calcium switch (+Ca^2+^) were treated for 40 min with 5 µM SBI-0206965. Whole cell lysates were analyzed for phosphorylation of AMPK at Thr-172 and ACC at Ser-79. Expression of β-actin served as loading control. (**B**) Time course of TEER development in WT Caco-2 cells subjected to a calcium switch in the presence or absence of the AMPK inhibitor SBI-0206965. Cells grown on Transwell filters were incubated in calcium-free medium for 16 hours and switched to normal calcium medium in the presence or absence of the AMPK inhibitor SBI-0206965. TEER was measured at the indicated time points after calcium switch and is given as fold change relative to the value in calcium-free medium (0 h time point). Data represent means ± SD (*n* = 3). * *p* < 0.05; ** *p* < 0.01; *** *p* < 0.005 versus SBI-0206965 treated cells for the same time point. # *p* <0.05 versus 0 h time point (0 h) for the same condition. (**C**) Cell lysates from WT Caco-2 cells at steady-state (basal) or subjected to calcium switch (Ca^2+^ switch) and treated for 40 min with or without 5 µM SBI-0206965 were blotted with anti-ZO-1 antibodies. Expression of β-actin served as loading control.

AMPK-dependent effect on the establishment of TEER during the calcium switch assay was also confirmed by incubating WT Caco-2 cells with SBI-0206965, a specific AMPK inhibitor [21]. Upon readdition of calcium, increased phosphorylation of AMPK and ACC, reflecting AMPK activity during TEER establishment, was fully abolished by treatment with SBI-0206965 (Figure 4A). Pharmacological inhibition of AMPK led to a significant inhibition of TEER establishment (Figure 4B) that was associated with impaired increase in protein levels of the tight junction-associated protein zonula occludens 1 (ZO-1) following calcium switch (Figure 4C). To exclude the possibility that SBI-0206965 acts via off-target effects, independently of AMPK inhibition, we evaluated its effect in AMPK dKO cells by measuring the development of TEER after calcium switch. SBI-0206965 had no effect on TEER (Supplementary Figure S2A) and ZO-1 protein levels were globally low (Supplementary Figure S2B) in the absence of AMPK, confirming the importance of AMPK during the re-establishment of TEER.

### 2.5. Pharmacological AMPK Activation Enhances the Reassembly of Tight Junctions

**Figure 5:**
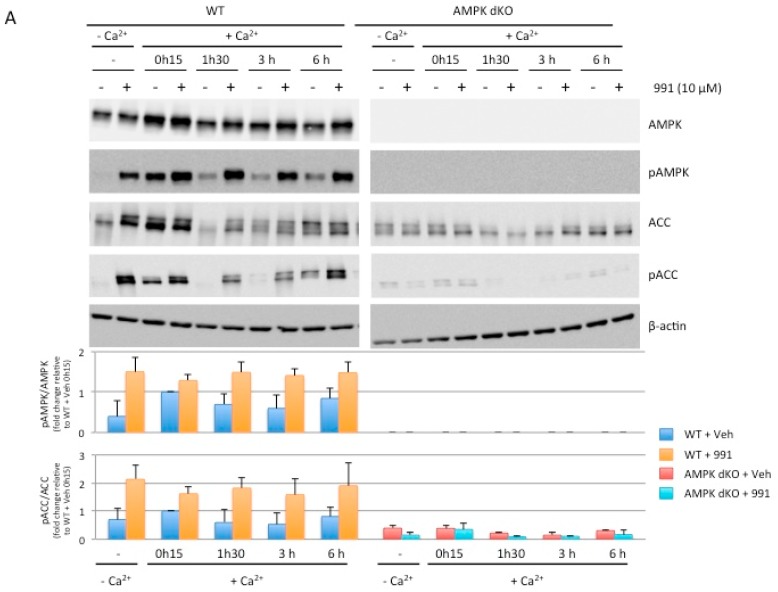

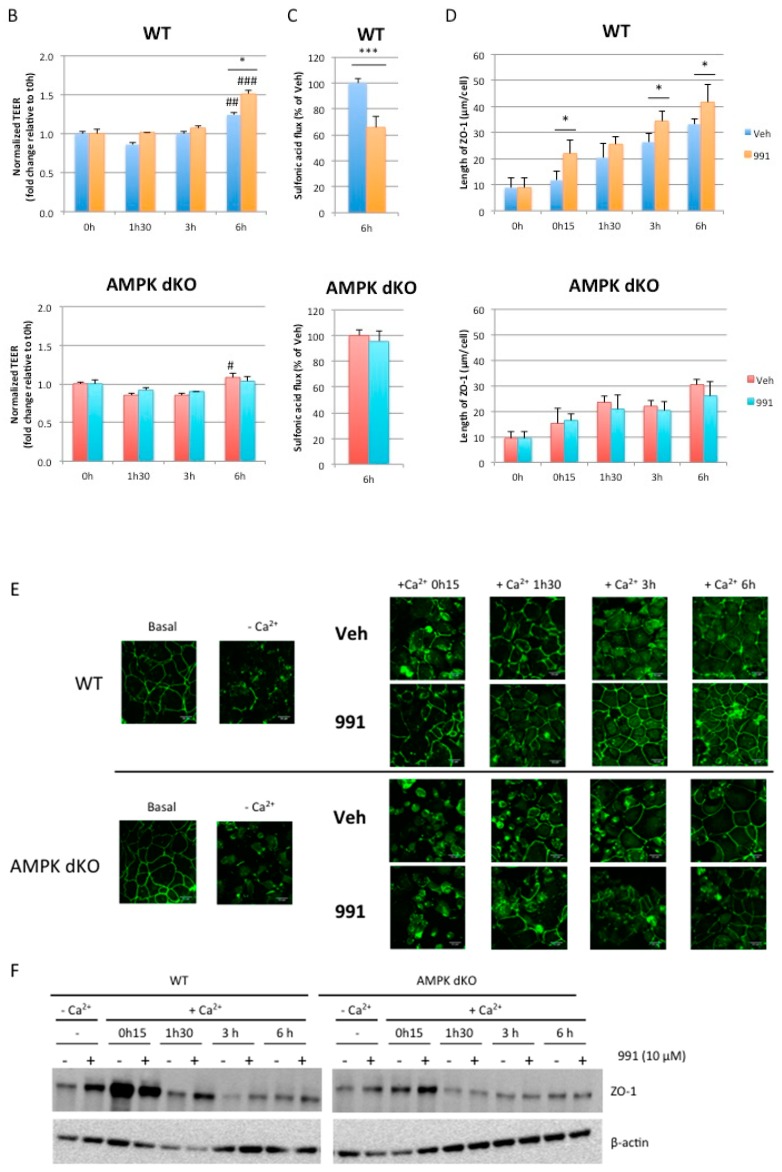
AMPK activation promotes tight junction assembly after calcium switch. (**A**) WT and AMPK dKO Caco-2 cells were subjected to calcium switch and were treated with DMSO or 10 µM 991 at indicated time points. Cell lysates were analyzed for Thr-172 phosphorylation and total AMPK expression, Ser-79 phosphorylation and total ACC expression by Western blotting. Expression of β-actin served as loading control. Lower panels represent ratios of pAMPK:AMPK and pACC:ACC from quantification of immunoblot images. (**B**) Time course of TEER development in WT and AMPK dKO Caco-2 cells subjected to a calcium switch in the presence or absence of the AMPK activator 991. Cells grown on Transwell filters were incubated in calcium-free medium for 16 hours and switched to normal calcium medium in the presence or absence of 10 µM 991. TEER was measured at the indicated time points after calcium switch and is given as fold change relative to the value in calcium-free medium at 0 h time point (0 h). (**C**) Paracellular permeability of 0.4 kDa sulfonic acid in WT and AMPK dKO Caco-2 monolayers subjected to calcium switch in the presence or absence of the AMPK activator 991. Paracellular flux was measured at the 6 h time point (6 h) and is given as the percentage of WT value. (**D**) Quantification of ZO-1 deposition at cell-cell junction in WT and AMPK dKO Caco-2 cells after calcium switch and treatment with or without 10 µM 991 at the indicated time points. (**E**) Representative immunostaining of ZO-1 in WT and AMPK dKO Caco-2 cells at steady-state (basal), incubated in calcium-free medium for 16 hours (-Ca^2+^) or subjected to calcium switch (+Ca^2+^) in the presence or absence of the AMPK activator 991 for indicated time. Scale bar: 25 µm. (**F**) After calcium switch, cell lysates were analyzed for ZO-1 expression by Western blotting. Expression of β-actin served as loading control. Data represent means ± SD for three independent experiments (*n* = 3). * *p* < 0.05; *** *p* < 0.005 versus 991 treated cells at the same time point. # *p* <0.05; ## *p* < 0.01; ### *p* < 0.005 versus 0 h time point (0 h) for the same condition.

We next investigated the effect of AMPK activation on the reassembly of tight junctions by treating Caco-2 cells with the pan-AMPK activator 991 upon readdition of calcium during the calcium switch assay. To confirm the effect of 991 on AMPK activation, we monitored the phosphorylation of AMPK and ACC during the experiment. Western blot data showed increased levels of AMPK and ACC phosphorylation in WT cells in response to 991 treatment in the absence of calcium (Supplementary Figure S3A) and during the calcium switch experiment at 1 h and 30 min, 3 h and 6 h; the effect of 991 was lost in AMPK dKO cells (Figure 5A). In the presence of 991, the establishment of TEER was significantly increased at 3 h and 6 h in WT cells whereas no effect was observed in AMPK dKO cells (Figure 5B). In accordance with the values of TEER, the paracellular permeability measured with the 0.4 kDa FITC-conjugated sulfonic acid was significantly decreased at 6 h in WT cells treated with 991, but not in AMPK dKO cells (Figure 5C). Consistent with these findings, we found that 991-induced AMPK activation in WT cells enhanced the reassembly of tight junction as revealed by the obvious changes in the morphological organization of tight junction by electron microscopy analysis (Supplementary Figure S4). We also monitored the effect of 991 on the location of ZO-1 during the calcium switch by immunofluorescence. We found that 991 induced a more rapid relocalization of ZO-1 at plasma membrane in WT cells, the quantification of ZO-1 deposit was significantly higher at 0 h and 15 min, 3 h, and 6 h compared to the control condition (Figure 5D,E). Furthermore, we showed that 991-induced AMPK activation had a positive effect on ZO-1 location even in the absence of calcium (Supplementary Figure S3B,C). Compound 991 had no effect on ZO-1 location in AMPK dKO cells (Figure 5D,E), in accordance with the lack of changes on TEER and paracellular permeability (Figure 5B,C). At protein expression level, the readdition of calcium induced an increase of ZO-1 at 0 h and 15 min in both conditions in the presence or in the absence of 991, but the effects on AMPK dKO cells were lower (Figure 5F). However, at later time points, the presence of 991 led to increased levels of the tight junction protein ZO-1 at 1 h and 30 min compared to the control condition in WT cells, and there was no effect in AMPK dKO cells (Figure 5F). These data indicate that AMPK participates in both the reassembly and maintenance of tight junctions over time. Thus, pharmacological activation could ensure a better recovery of intestinal integrity after disruption of barrier function.

## 3. Discussion

In the current study, we highlighted the importance of AMPK in the initiation of tight junctions reassembly following a disruption of the intestinal epithelial barrier. We used Caco-2 cell monolayers as an *in vitro* model of the intestinal barrier and manipulated the assembly of tight junctions in calcium switch assays, which have been widely used for studying the barrier competence of the intestinal epithelium. To study the role of AMPK in the assembly of tight junctions, we generated a Caco-2 model invalidated for both isoforms of the catalytic subunit of AMPK (AMPKα1 and AMPKα2). The relevance to work with AMPK dKO Caco-2 cells was justified by the increased expression of the non-deleted AMPKα2-isoform when only the predominant AMPKα1 isoform was knocked-out. In addition, we observed that pharmacological activation of AMPK in AMPKα1 KO Caco-2 cells triggered phosphorylation of ACC to similar levels than in WT cells, raising the possibility that each AMPKα-isoform can substitute for the other, with respect to both phosphorylation of downstream targets and amelioration of tight junction assembly. However, further work is warranted to elucidate potential AMPK isoform-specific role in the regulation of intestinal barrier function.

In steady-state conditions, when Caco-2 cells are fully confluent and polarized on Transwell filters, AMPK does not seem to play a crucial role in the maintenance of tight junctions, as values of TEER were similar between WT and AMPK dKO cells. In line with these results, previous study has reported that AMPK was not necessary for the maintenance of tight junctions in confluent MDCK cells [12]. Although there were no obvious differences in the epithelial barrier function and the ultrastructure of tight junctions between WT and AMPK dKO cells at steady-state, we found that AMPK dKO cells showed impaired development of TEER when tight junctions integrity was challenged during a calcium switch assay. Upon readdition of calcium, AMPK dKO cells displayed a delay in the re-establishment of the epithelial barrier, as shown by a slower establishment of TEER and relocalization of ZO-1 at plasma membrane, and a higher paracellular permeability compared to WT cells. The use of the AMPK inhibitor SBI-0206965 also confirmed a role for AMPK in the re-establishment of TEER and in the reassembly of tight junctions during the calcium switch assay. Thus, inhibition of AMPK either genetically or chemically was associated with impairment in the restoration of epithelial barrier integrity, demonstrating the modulatory role of AMPK in intestinal epithelial barrier function. Although our results do not permit to conclude on the requirement of AMPK in the initiation of tight junction assembly, they indicate that AMPK is necessary but not sufficient in this process. AMPK dKO cells are still able to induce the formation of tight junction, meaning that these cells have probably developed compensatory mechanisms to balance the absence of AMPK.

As already reported in MDCK cells [11], we observed an increase in the activation of AMPK upon the readdition of calcium in Caco-2 cells, as revealed by enhanced AMPK and ACC phosphorylation in this context. We hypothesized that treatment with calcium could lead to the activation of CaMKKβ, a well-known upstream kinase for AMPK [9], which in turn could phosphorylate and activate AMPK. AMPK activation through CaMKK but not LKB1 was also suggested in MDCK cells because levels of nucleotides were not significantly altered during the calcium switch assay [11], and variations in cellular energy status rather tend to facilitate activation of AMPK by the upstream kinase LKB1 [27]. However, a study conducted on Caco-2 cells showed that the absence of LKB1 could induce impairments in polarization. It has notably been found that LKB1 was important for ZO-1 location at plasma membrane [28]. Thus, further investigation will be required to better understand the role of upstream AMPK kinases in the AMPK-mediated regulation of tight junction assembly.

In previous studies, it has been shown that epithelial AMPK activation with the commonly used pharmacological AMPK activator, AICAR, was closely associated with enhanced tight junction integrity in MDCK cells during calcium switch [11,12]. The authors suggested that these effects could be mediated by the activation of AMPK. Treatment with AICAR also improved epithelial barrier function in Caco-2 cells as shown by increased TEER and decreased paracellular permeability of FITC-dextran [19,29]. However, the specificity of AICAR has been questioned due to its ability to activate AMP-dependent enzymes and is regarded as a non-specific activator of AMPK [30]. Here, we analyzed the impact of AMPK activation in Caco-2 cells by using a novel class of direct allosteric AMPK activators, binding to the allosteric drug and metabolite (ADaM) site, located between the α-subunit kinase domain and β-subunit carbohydrate binding module of AMPK complex [31]. Here, we showed that activation of AMPK with the pan-AMPK activator 991 facilitated the reassembly of tight junctions by increasing TEER and decreasing the paracellular permeability during the calcium switch assay. Compound 991 also induced a faster relocalization of ZO-1 at cell-cell contact and maintained expression levels of ZO-1. Importantly, all the beneficial effects of 991 were lost in AMPK dKO cells showing AMPK-dependent action. Interestingly, compound 991 also had a beneficial effect on the initiation of tight junctions reassembly in calcium-free medium, meaning that the sole AMPK activation is sufficient to induce this process. However, the combination of compound 991 and calcium enables a greater impact on the epithelial barrier recovery associated with enhanced AMPK activation over time.

How AMPK activation promotes the assembly of tight junction proteins remains to be elucidated. It has been reported that rapamycin rescues the delay of tight junction assembly in MDCK cells expressing an AMPK kinase-dead mutant, indicating that AMPK may act, at least partially, through the regulation of mammalian target of rapamycin (mTOR) signaling [11]. AMPK may also directly act on tight junctions as it has been revealed in a phosphoproteomic study that ZO-2, another protein belonging to the zonula occludens family, could be a potential target of AMPK [32]. Otherwise, it could also be possible that AMPK phosphorylates proteins involved in the regulation of tight junctions, such as proteins kinase C (PKC), initially defined as signaling proteins. Indeed, some PKC isoforms are localized at plasma membrane and participate in the maintenance and the assembly of tight junctions. In particular, both PKCη and PKCζ play a key role in the localization of ZO-1 and occludin at the membrane and are able to phosphorylate occludin [33,34]. A study performed in Caco-2 cells reported that AMPK activation by sodium butyrate led to PKCβ phosphorylation, which is necessary to ensure a correct recovery of the epithelial barrier during a calcium switch assay [35]. In the same study, the authors also found that AMPK activation regulated the phosphorylation of myosin II regulatory light chain (MLC2) by decreasing the activity of MLCK, involved in the contraction of the cytoskeleton and the regulation of paracellular permeability. Interestingly, it has been shown that AMPK directly phosphorylates MLC2 in *Drosophila* and knock-out of AMPK in fly causes abnormal epithelial integrity [36]. It has also been reported that AMPK could facilitate adherens junctions formation by the phosphorylation of afadin to regulate its interaction with the tight junction-associated protein ZO-1, thereby facilitating ZO-1 distribution to the plasma membrane [37,38].

Lastly, two new AMPK substrates recently identified could be of interest to explain the action of AMPK activation on the reassembly of tight junctions. First, cingulin, a protein involved in the association of planar apical microtubules with tight junctions, has been found by a motif affinity proteomic approach designed for the discovery of AMPK substrates in primary mouse hepatocytes [32]. AMPK-mediated phosphorylation of cingulin at Ser137 was validated in Caco-2 cells treated with 991 [32]. The importance of cingulin in the association between microtubules and tight junctions was further revealed in mouse mammary epithelial Eph4 cells by using a knockdown model [39]. Second, a novel effector of AMPK has been discovered in MDCK cells. Under stress conditions, AMPK was shown to phosphorylate the G-alpha interacting vesicle associated protein (GIV), a scaffold protein involved in cell polarity. GIV is notably able to bind aPKC/Par3/Par6 and cadherin/catenin complexes. Thus, the AMPK-GIV signaling pathway stabilizes tight junction proteins [40]. AMPK-mediated phosphorylation of GIV at Ser245 has been recently confirmed by using AMPK-deficient enteroids derived monolayers treated with metformin, another commonly used indirect AMPK activator [41]. However, future studies determining the role of GIV and cingulin phosphorylation in the mechanisms mediating AMPK effects on tight junction are required.

In conclusion, we demonstrated that pharmacological AMPK activation has the ability to enhance the assembly of tight junction in intestinal Caco-2 cells, likely through a faster relocalization of ZO-1 at cell-cell contact. In addition to pharmacological drugs, SCFAs [19,42] as well as many naturally occurring phytochemicals [43,44,45,46] have been shown to improve barrier function associated with AMPK activation. Thus, our data provide support for targeting AMPK by the use of pharmacological, as well as nutraceutical compounds, to strengthen intestinal epithelial barrier functions and to exert health beneficial effects on leaky gut, predisposing individuals to intestinal bowel disease, obesity and diabetes.

## 4. Materials and Methods 

### 4.1. Reagents and Antibodies

Compound 991 (5-{[6-chloro-5-(1-methylindol-5- yl)-1H- benzimidazol-2- yl] oxy}-2- methyl- benzoic acid; CAS no. 129739-36-2) was synthesized by Spirochem (Spirochem, Zürich, Switzerland). SBI-0206965 was purchased from Sigma-Aldrich (#SML 1540, Sigma-Aldrich, Saint-Quentin-Fallavier, France). FITC-conjugated sulfonic acid (Fluorescein-5-(and-6)-Sulfonic acid) was obtained from ThermoFisher Scientific (#F1130, ThermoFisher Scientific, Waltham, MA, USA). Primary antibodies directed against total AMPKα (#2532), AMPKα phosphorylated at Thr-172 (#2531), total acetyl-CoA carboxylase 1/2 (ACC1/2) (#3676), ACC phosphorylated at Ser-79 on ACC (#3661), and β-actin (#4967) were purchased from Cell Signaling Technology (CST, Danvers, MA, USA). ZO-1 antibodies were obtained from Cell Signaling Technology (#13663, CST, Danvers, MA, USA) and ThermoFisher Scientific (#33-9100, ThermoFisher Scientific, Waltham, MA, USA). Anti-AMPKα1 and anti-AMPKα2 antibodies were kindly donated by Grahame Hardie (University of Dundee, Dundee, UK). HRP-conjugated secondary antibodies, goat anti-mouse IgG (#401215) and goat anti-rabbit IgG (#401393) were purchased from Calbiochem (EMD Chemicals Inc., San Diego, USA and EMD Millipore Corp., Billerica, USA, respectively) and Alexa Fluor 488-conjugated anti-rabbit IgG antibodies were obtained from ThermoFisher (#A-11008, ThermoFisher Scientific, Eugene, OR, USA). 

### 4.2. Generation of AMPK Deficient Caco-2 Cells using CRISPR-Cas9 Gene Editing System

Generation of AMPKα1/α2 KO Caco-2 cells was previously described [23]. Briefly, guide RNA sequences for targeting *PRKAA1* (encoding AMPKα1) and *PRKAA2* (encoding AMPKα2) genes were designed by using the online CRISPR design tool at http://crispr.mit.edu. Single guide RNA (sgRNA) sequences for AMPKα1 and AMPKα2 were as follows: 5′-AGGGCACGCCATACCCTTG-3′ and 5′-AAGATCGGACACTACGTGCT-3′, respectively. The non-targeted scramble guide sequence: 5′-GTAGGCGCGCCGCTCTCTAC-3′ was used as control. Each sgRNA was cloned into pLentiCrispr plamids (Addgene #52961 and #57818) and lentivirus were produced in HEK293-T packaging cells. Caco-2 cells were first infected with lentivirus expressing *PRKAA1* sgRNA, and selected with puromycin. Single cell cloning was performed by cell sorting into 96-well plates and individual clones were screened by Western blot analysis using AMPKα1 antibodies. A single-cell AMPKα1-deficient clone was then infected with lentivirus expressing *PRKAA2* sgRNA, and cells were sorted based on the expression of GFP. Single colonies were screened by Western blot using AMPKpanα, and screened for insertion and deletion (indel) mutation after amplification of PCR products of target *PRKAA2* gene with Phire hot start DNA polymerase (ThermoFisher Scientific, Life Technologies SAS, Courtabœuf, France) using primers 5′-GCTGCACTGTGGGTAGGC-3′ and 5‘-GGGCGTCGGCACCTTC-3′. Indel mutations were validated by DNA sequencing analysis and a clone lacking both AMPK α1 and α2 protein expression was selected.

### 4.3. Cell Culture and Calcium Switch Assay

Caco-2 cells were obtained from American Type Culture Collection (ATCC) and were maintained at 37 °C with 5% CO_2_ and 95% air atmosphere. Cells were grown in MEM (ThermoFisher Scientific, Life Technologies SAS, Courtabœuf, France) supplemented with FBS 20% (Eurobio), penicillin/streptomycin 1%, and non essential amino acids 1% (ThermoFisher Scientific, Life Technologies SAS, Courtabœuf, France). 

Caco-2 cells were grown for 21 days on permeable filters with 8 µm pore diameter (Greiner Bio-One, Courtabœuf, France). Polarized cells were washed twice with calcium-free Minimum Essential Medium (SMEM) and then incubated with SMEM supplemented with calcium-free FBS 3% for 16 h. SMEM was removed, cells were washed with MEM, then cells were incubated with MEM for 6 h or 10 h. Depending on experimental conditions, cells could be treated with 10 µM 991 or 5 µM SBI-0206965 during re-incubation with regular MEM. Barrier integrity was monitored over time by measurements of TEER and paracellular permeability. 

### 4.4. TEER and Paracellular Permeability Measurements

TEER was measured with an epithelial volt-ohmmeter (World Precision Instruments, Sarasota, FL, USA), for each well 3 measures were performed. During calcium switch assay, TEER was measured before incubation with SMEM, and then at 0 h, 1 h and 30 min, 3 h, 6 h, and 10 h after readdition of regular MEM. 

Paracellular permeability was measured at 6 h after readdition of regular MEM with Fluorescein-5-(and-6)-Sulfonic acid 0.4 kDa (1g/L). 50 µL of FITC-sulfonic acid were added into the apical part and was incubated for 1h at 37 °C. Then 150 µL of medium from the basal part were collected in a microplate, and the fluorescence was measured at an excitation wavelength of 485 nm and an emission wavelength of 520 nm with FLUOstar OPTIMA microplate reader (BMG Labtech, Champigny-sur-Marne, France).

### 4.5. Immunofluorescence and Quantification of ZO-1

Cells on filters were fixed in cold methanol for 5 min at -20 °C and blocked with BSA 10% for 1h at room temperature. Cells were then incubated with anti-ZO-1 antibodies from Cell Signaling Technology (CST, Danvers, MA, USA) overnight at 4 °C, followed by incubation with Alexa Fluor 488-conjugated anti-rabbit IgG (ThermoFisher Scientific, Eugene, Oregon, USA). Cell nuclei were stained with Hoechst. Mowiol was used as the mounting medium. Cells were observed on Spinning Disk confocal microscope, and pictures were obtained from MetaMorph software (Molecular Devices, San Jose, CA, USA). 

Quantification of ZO-1 length was calculated by outlining manually ZO-1 staining at plasma membrane at cell junctions on six areas randomly chosen in each coverslip as previously described [11]. Outlined length were measured on ImageJ software (National Institutes of Health, Bethesda, MD, USA) and then divided by nuclei number in each area to get the average ZO-1 length per cell. 

### 4.6. Electron Microscopy

Cells on filters were fixed with 3% glutaraldehyde in phosphate buffer 0.2 M (pH 7.2) and were then embedded in epoxy resin. Samples were examined with a transmission electron microscope (JEOL 1011, JEOL Europe SAS, Croissy-sur-Seine, France) at the Cochin Institute electron microscopy facility using a GATAN eslangshen camera and the Digital Micrograph software (Gatan Microscopy Suite, Gatan Inc., Pleasanton, CA, USA).

### 4.7. Western Blotting

Cells were lysed in ice-cold lysis buffer containing 50 mM Tris-HCl, pH 7.4, 1% Triton X-100, 150 mM NaCl, 1 mM EDTA pH 7.4, 1mM DTT, 10% glycerol, and protease (Roche Complete Protease Inhibitor Cocktail, Sigma-Aldrich, Saint-Quentin-Fallavier, France) and phosphatase (Pierce phosphatase inhibitor, ThermoFisher Scientific, Life Technologies SAS, Courtabœuf, France) inhibitors. Lysates were sonicated on ice for 4 × 30 s to shear DNA and reduce viscosity. The lysates were centrifuged for 5 min at 10,000× *g* at 4 °C, and the supernatants were collected for the determination of total protein content with a BCA protein assay kit (ThermoFisher Scientific, Life Technologies SAS, Courtabœuf, France). Supernatant proteins (20 μg) were separated by SDS-PAGE in precast 4-15% polyacrylamide gels and the resulting bands were transferred to nitrocellulose membranes. Equal loading was checked by Ponceau Red staining of the blot membrane before blocking by incubation for 30 min at room temperature with Tris-buffered saline supplemented with 0.2% NP40 and 5% non-fat dry milk. Total and phosphorylated AMPK and ACC, ZO-1, and β-actin were probed from separated membranes. Immunoblotting was performed according to standard procedures, and the signals were detected with chemiluminescence reagents (EMD Chemicals Inc., San Diego, USA and EMD Millipore Corp., Billerica, USA) by using ImageQuant LAS 4000 (GE Healthcare). Band intensities were quantified by Image J (National Institutes of Health, Bethesda, MD, USA) densitometry analysis and ratios for pAMPK:AMPK and pACC:ACC were calculated relative to the control condition.

### 4.8. Statistical Analysis

Results are expressed as means ± SD. Groups were compared in unpaired two-tailed Student’s *t*-tests, one-way ANOVA with Dunnett’s *post hoc* test for longitudinal and one single time point data comparisons or two-way ANOVA with Bonferroni’s *post hoc* test for multiple comparisons as appropriate with GraphPad Prism 5.0 (GraphPad Software Inc., San Diego, CA, USA). Differences between groups were considered statistically significant if *p* < 0.05.

## Supplementary Materials

Supplementary materials can be found at https://www.mdpi.com/1422-0067/20/20/5171/s1.

## Abbreviations

ACC	Acetyl CoA carboxylase
ADaM	Allosteric drug and metabolite
AMPK	AMP-activated protein kinase
CaMKKβ	Ca^2+^ calmodulin-activated protein kinase kinase beta
CRISPR/Cas9	Clustered regularly interspaced short palindromic repeats/associated protein 9 endonuclease
FITC	Fluorescein-5-isothiocyanate
GFP	Green fluorescent protein
GIV	G-alpha interacting vesicle associated protein
LKB1	Liver kinase B1
MDCK	Madin-Darby canine kidney
MLC2	Myosin II regulatory light chain
mTOR	Mammalian target of rapamycin
PKC	Proteins kinase C
SCFAs	Short chain fatty acids
TEER	Trans-epithelial electrical resistance
ZO-1	Zonula occludens 1
