# Supplementary material for: AMPK Activation Promotes Tight Junction Assembly in Intestinal Epithelial Caco-2 Cells
Source: Int J Mol Sci. 2019 Oct 18;20(20):5171. doi: 10.3390/ijms20205171 (PMC6829419; doi:10.3390/ijms20205171)
Supplement: Supplementary file 1 [file ijms-20-05171-s001.pdf]

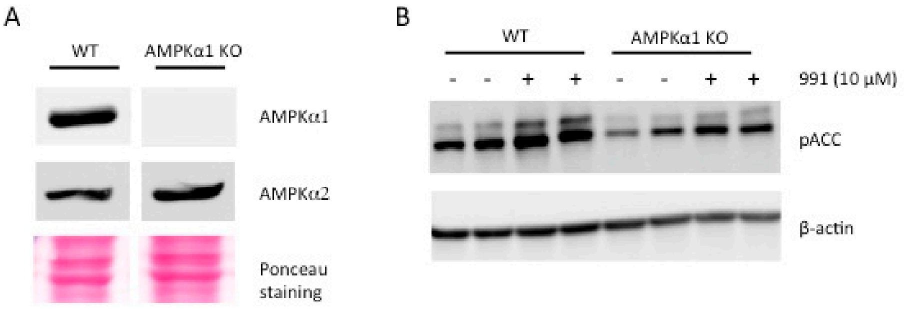

Suppl Figure S1: Characterization of AMPKα1-deficient Caco2 cells. (A) Western blot analysis of AMPKα1 and AMPKα2 protein abundance in Caco-2 cells expressing non-targeted (WT) or *PRKAA1*-targeted sgRNAs (AMPKα1 KO). Ponceau staining served as loading control. (B) Whole cell lysates of WT and AMPKα1 KO Caco-2 cells treated with 10 μM 991 were analyzed for the phosphorylation of ACC at Ser79. Expression of β-actin served as loading control.

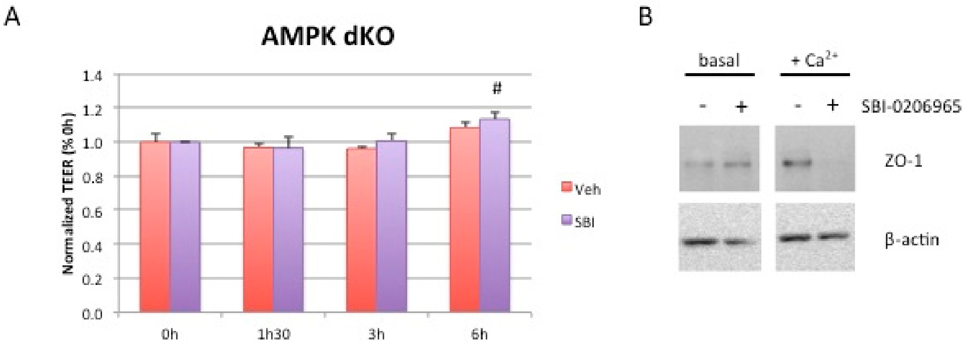

Suppl Figure S2: Effect of acute pharmacological inhibition of AMPK in AMPK dKO Caco-2 cells on establishment of TEER after calcium switch. (A) Time course of TEER development in AMPK dKO Caco-2 cells subjected to a calcium switch in the presence or absence of the AMPK inhibitor SBI-0206965. Cells grown on Transwell filters were incubated in calcium-free medium for 16 hours and switched to normal calcium medium in the presence or absence of 5 μM SBI-0206965. TEER was measured at the indicated time points after calcium switch and is given as fold change relative to the value in calcium-free medium at 0 hour time point (t0h). Data represent means ± SD for three independent experiments (n=3). #$p<0.05$ versus 0 hour time point (t0h) for the same condition. (B) Cell lysates from AMPK dKO Caco-2 cells at steady-state (basal) or subjected to

calcium switch (Ca^{2+} switch) and treated for 40 min with or without 5 μM SBI-0206965 were blotted with anti-ZO-1 antibodies. Expression of β-actin served as loading control.

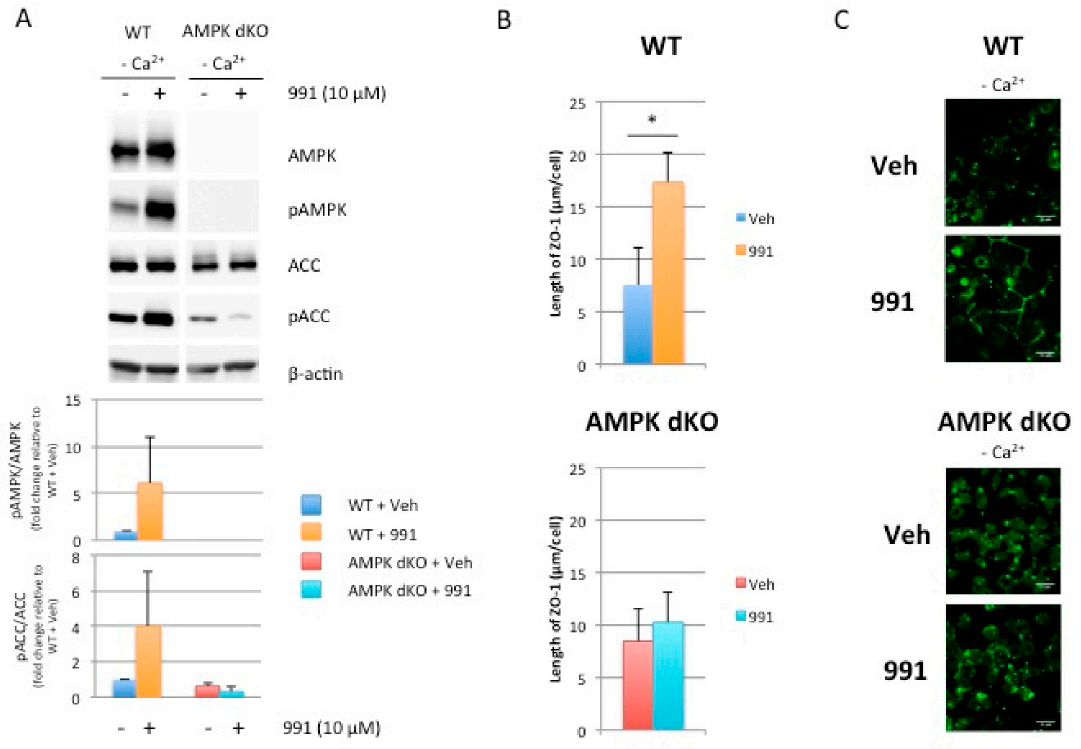

Suppl Figure S3: AMPK activation promotes ZO-1 location at plasma membrane in the absence of calcium. (A) Cells grown on Transwell filters were incubated in calcium-free medium for 16 hours and treated with or without 10 μM 991 for 15 min. Cell lysates were analyzed for Thr-172 phosphorylation and total AMPK expression, Ser-79 phosphorylation and total ACC expression by western blotting. Expression of β-actin served as loading control. Lower panels represent ratios of pAMPK:AMPK and pACC:ACC from quantification of immunoblot images. (B) Quantification and (C) representative immunostaining of ZO-1 deposition at cell-cell junction in WT and AMPK dKO Caco-2 cells incubated in calcium-free medium for 16 hours and treated with or without 10 μM 991 for 15 min.

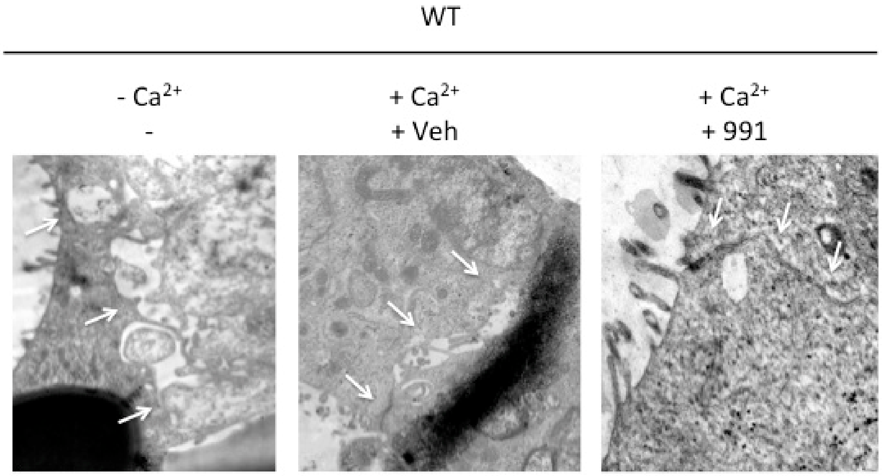

Suppl Figure S4: Effect of AMPK activation in Caco-2 cells on tight junction ultrastructure during calcium switch. Transmission electron microscopy ultrastructure of tight junction of monolayers of WT Caco-2 cells incubated in calcium-free medium, after calcium switch in the presence or absence of 10 μM 991 for 6 hours Scale bar is 200 nm. Arrows indicate the localization of cell-cell junctions.
